# Rib fixation in patients with severe rib fractures and pulmonary contusions: Is it safe?

**DOI:** 10.1097/TA.0000000000003790

**Published:** 2022-09-19

**Authors:** Suzanne F.M. Van Wijck, Fredric M. Pieracci, Elizabeth F. Smith, Kelley Madden, Ernest E. Moore, Mathieu M.E. Wijffels, Nicole L. Werner

**Affiliations:** From the Department of Surgery (S.F.M.V.W., F.M.P., E.F.S., K.M., E.E.M., N.L.W.), Ernest E Moore Shock Trauma Center at Denver Health, Denver, Colorado; and Trauma Research Unit Department of Surgery (S.F.M.V.W., M.M.E.W.), Erasmus MC, University Medical Center Rotterdam, Rotterdam, the Netherlands.

**Keywords:** Pulmonary contusion, thoracic trauma, rib fracture, SSRF, outcomes

## Abstract

Pulmonary contusion has been considered a contraindication to surgical stabilization of rib fractures (SSRFs). This study aimed to evaluate the association between pulmonary contusion severity and outcomes after SSRF. We hypothesized that outcomes would be worse in patients who undergo SSRF compared with nonoperative management, in presence of varying severity of pulmonary contusions.

**METHODS:**

This retrospective cohort study included adults with three or more displaced rib fractures or flail segment. Patients were divided into those who underwent SSRF versus those managed nonoperatively. Severity of pulmonary contusions was assessed using the Blunt Pulmonary Contusion 18 (BPC18) score. Outcomes (pneumonia, tracheostomy, mechanical ventilation days, intensive care unit (ICU) length of stay, hospital length of stay, mortality) were retrieved from patients' medical records. Comparisons were made using Fisher's exact and Kruskal-Wallis tests, and correction for potential confounding was done with regression analyses.

**RESULTS:**

A total of 221 patients were included; SSRF was performed in 148 (67%). Demographics and chest injury patterns were similar in SSRF and nonoperatively managed patients. Surgical stabilization of rib fracture patients had less frequent head and abdominal/pelvic injuries (*p* = 0.017 and *p* = 0.003). Higher BPC18 score was associated with worse outcomes in both groups. When adjusted for ISS, the ICU stay was shorter (adjusted *β*, −2.511 [95% confidence interval, −4.87 to −0.16]) in patients with mild contusions who underwent SSRF versus nonoperative patients. In patients with moderate contusions, those who underwent SSRF had fewer ventilator days (adjusted *β*, −5.19 [95% confidence interval, −10.2 to −0.17]). For severe pulmonary contusions, outcomes did not differ between SSRF and nonoperative management.

**CONCLUSION:**

In patients with severe rib fracture patterns, higher BPC18 score is associated with worse respiratory outcomes and longer ICU and hospital admission duration. The presence of pulmonary contusions is not associated with worse SSRF outcomes, and SSRF is associated with better outcomes for patients with mild to moderate pulmonary contusions.

**LEVEL OF EVIDENCE:**

Therapeutic/Care Management; Level IV.

## Abstract 2

Pulmonary contusions have been considered a contra-indication to rib fixation. However, pulmonary contusions are not associated with worse rib fixation outcomes, and it might even be associated with better outcomes for patients with mild to moderate pulmonary contusions. #ssrf.

## Graphical abstract

**Figure:**
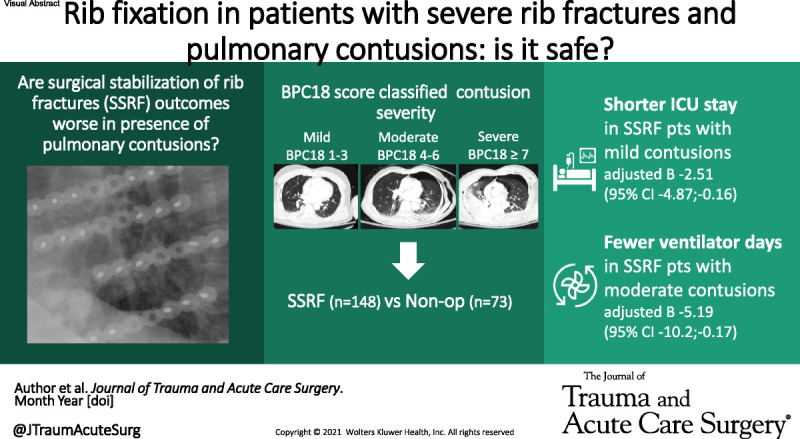

Chest wall trauma is prevalent and morbid. Rib fractures account for around 10% of all trauma admissions and are associated with increased mortality and morbidity, and decreased quality of life.^1–6^ Severe rib fracture patterns are often accompanied by other injuries, especially pulmonary contusions.^7,8^ Pulmonary contusions are documented in more than 50% of patients with a flail chest.^9^ Pulmonary contusions have dynamic pathophysiology, which, depending on the extent of the injury, can lead to pneumonia, acute respiratory distress syndrome, and mortality.^8,10,11^

Currently, chest computed tomography (CT) is the recommended diagnostic modality, since it is highly sensitive in diagnosing pulmonary contusions.^12,13^ Because pulmonary contusions can vary in size and severity, dichotomously classifying the presence or absence of pulmonary contusions is insufficient to appreciate the extent of the injury.^14^ Furthermore, the severity of the pulmonary contusions detected on CT scan can change radiographically over time, especially in the first hours after injury.^15,16^

Surgical stabilization of rib fractures (SSRFs) is increasingly used in the management of patients with severe rib fractures.^17^ Pulmonary contusions have traditionally been considered a relative contraindication to SSRF because the pulmonary morbidity is presumed to arise predominantly from the contusion as opposed to the rib fractures.^18–20^ Moreover, pulmonary contusion may increase the risk of general anesthesia and SSRF. However, there is a paucity of published data specifically about SSRF concerning the presence and severity of pulmonary contusions.

This study aimed to evaluate the association between pulmonary contusion severity and outcomes after SSRF. We hypothesized that outcomes would be worse in patients who undergo SSRF compared with patients whose rib fractures are managed nonoperatively.

## PATIENTS AND METHODS

### Setting and Study Population

We retrospectively analyzed trauma patients who were admitted with three or more displaced (≥50% cortical displacement on axial CT imaging) rib fractures or flail segment (two or more consecutive ribs with fractures in two or more locations) from our prospectively maintained database in a level 1 trauma center. We included adult (18 years or older) patients from October 2010 to October 2021 if a chest CT was conducted on the first day of admission. Approval from the institutional review board was obtained. The Strengthening the Reporting of Observational Studies in Epidemiology (STROBE) guideline was used to ensure proper reporting of methods, results, and discussion (Supplemental Digital Content, Supplementary Data 1, http://links.lww.com/TA/C702).

### Variables

Clinical data were retrieved from the patient's medical records, including age, sex, past medical history, time and mechanism of injury, Abbreviated Injury Scale (AIS) score and Injury Severity Score (ISS), injury characteristics, and surgical procedures. Similarly, clinical outcomes were retrieved. The primary outcome variable studied was pneumonia rate. Secondary outcomes included rates of tracheostomy, mortality, mechanical ventilation days, and intensive care and hospital length of stay. All patients requiring mechanical ventilation were placed on a standard protocol including lung protective (6–8 mL/kg ideal weight) ventilation. Patients with severe head injury as defined by head AIS score of >3 were excluded from the analysis evaluating the tracheostomy rate. Patients were evaluated on the admission chest CT for the presence of hemothorax, pneumothorax, bilateral rib fractures, flail segment, the total number of rib fractures, RibScore, and fracture displacement (undisplaced, offset, displaced) as defined by the Chest Wall Injury Society taxonomy.^21,22^

### Pulmonary Contusions

**Figure 1:**
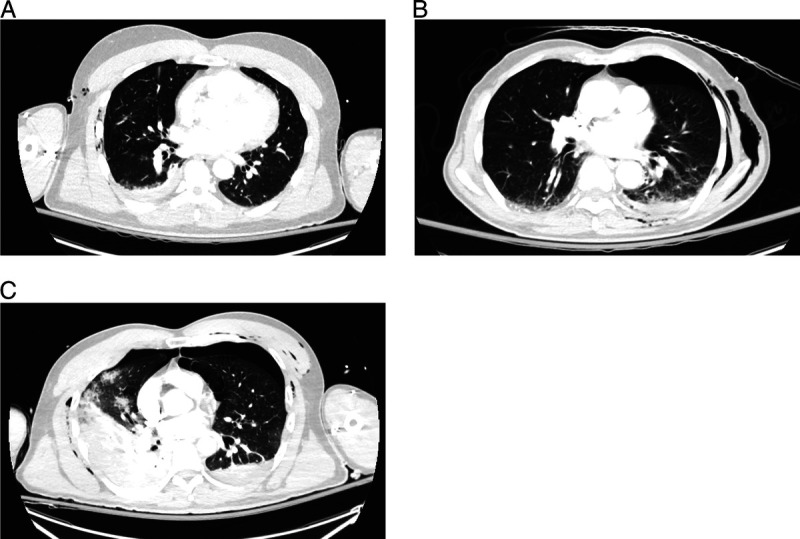
Examples of patients with mild, moderate, and severe pulmonary contusions on axial views of the admission chest CT: (*A*) upper left, mild contusion; (*B*) upper right, moderate contusion; and (*C*) lower left, severe contusion.

The presence and severity of pulmonary contusions were evaluated on the admission chest CT in axial and coronal views in the lung window on a maximum slice thickness of 3.5 mm. Pulmonary contusion severity was quantified using the Blunt Pulmonary Contusion 18 (BPC18) score.^15^ In this score, the lung fields are divided into an upper, middle, and lower third, and for each third, a score of 1 to 3 was assigned. A score of 1 corresponds with mild contusion with up to 33% opacification of the field, a score of 2 is a moderate contusion with 33% to 66% opacification, and a score of 3 corresponds to severe contusion with more than 66% opacification. The scores are summed, resulting in a maximum score of nine per lung, and a maximum total score of 18. All chest CTs were reviewed independently for BPC18 by at least two observers. One of the observers was a physician who reviewed and scored all chest CTs. The second independent BPC18 score was assigned either by another physician or a research coordinator who was trained by the other physicians to score BPC18. Cases with more than 3 points difference were reviewed again within the research team to reach consensus on the score. The BPC18 score assigned by the observer who scored all chest CTs was used when discrepancies of less than three points occurred. Pulmonary contusion severity was defined as mild with BPC18 scores of 1 to 3, moderate with 4 to 6, and severe with 7 to 18. An example of a mild, moderate, and severe pulmonary contusion is shown in Figure 1.

### Statistical Analysis

Data were analyzed using the Statistical Package for the Social Sciences version 28 (SPSS, Chicago, IL). The normality of continuous data was tested with the Shapiro-Wilk test. All continuous variables except age were nonparametric and are presented as median with percentiles. Categorical variables are presented as frequencies and percentages. Missing values were not replaced. Patients were divided into those who underwent SSRF versus those managed nonoperatively and stratified for pulmonary contusion severity. Comparisons were made using the independent *t* test and χ^2^ test for normally distributed data and the Mann-Whitney *U* and Fisher's exact tests for nonparametric data. Adjustment for confounding by concomitant injury was done with logistic regression analyses for the association between SSRF and categorical outcomes and linear regression for the continuous clinical outcomes. *p* Values were considered significant if below 0.05.

## RESULTS

A total of 221 patients were included, and SSRF was performed in 148 (67%). The mean age was 52 years in both with SSRF and nonoperatively managed patients (*p* = 0.812). Other baseline patient characteristics were also comparable between groups (Table 1). Nonoperatively managed patients had a similar chest injury severity to SSRF patients; both had a median chest AIS score of 3 (P_25_–P_75_, 3–4; *p* = 0.479). However, the nonoperatively managed patients were overall more severely injured with a trend toward a higher ISS (median, 24 [P_25_–P_75_, 14–35] vs. 21 [P_25_–P_75_, 17–27]; *p* = 0.068), and a significantly higher head AIS score (median, 0 [P_25_–P_75_, 0–3] vs. 0 [P_25_–P_75_, 0–2]; *p* = 0.017) and abdomen/pelvis AIS score (median, 2 [P_25_–P_75_, 0–3] vs. 0 [P_25_–P_75_, 0–2]; *p* = 0.003) compared with patients who underwent SSRF. The median time between injury and admission CT was just below 2 hours in both groups (*p* = 0.761). The BPC18 score on admission CT was 4 (P_25_–P_75_, 2–5, and P_25_–P_75_, 3–6; *p* = 0.144) in both nonoperatively managed and SSRF patients. Among the SSRF group, the median time to surgery was 1 day (P_25_–P_75_, 0–2).

**TABLE 1:** Baseline and Injury Characteristics of Patients Who Underwent SSRFS, Compared With Patients Who Were Managed Nonoperatively

	SSRF n = 148	Nonoperative n = 73	*p*
Age, mean (SD), y	52 (16)	52 (16)	0.812
Male	103 (70%)	52 (71%)	0.876
Comorbidities any	70 (47%)	37 (51%)	0.669
Asthma	12 (8%)	4 (5%)	0.588
COPD	6 (4%)	4 (5%)	0.733
Diabetes	19 (13%)	8 (11%)	0.828
Chronic kidney disease	0 (0%)	1 (1%)	0.330
Chronic heart failure	1 (1%)	0 (0%)	0.670
Current smoker	47 (32%)	28 (38%)	0.366
BMI	26 (23–30)	27 (24–31)	0.118
Injury mechanism MVC/MCC	60 (41%)	33 (45%)	0.145
Auto vs. pedestrian	27 (18%)	12 (16%)	
Auto vs. bike or ski accident	29 (20%)	8 (11%)	
Fall	29 (20%)	16 (22%)	
Crush injury	2 (1%)	0 (0%)	
Other or unknown	1 (1%)	4 (5%)	
ISS	21 (17–27)	24 (14–35)	0.068
AIS head/neck	**0 (0–2)**	**0 (0–3)**	**0.017**
Face	0 (0–0)	0 (0–0)	0.769
Chest	3 (3–4)	3 (3–4)	0.479
Abdomen/pelvis	**0 (0–2)**	**2 (0–3)**	**0.003**
Extremities	2 (0–2)	2 (0–2)	0.342
External	1 (0–1)	1 (0–1)	0.578
Isolated thoracic injury	14 (9%)	3 (4%)	0.190
Spinal fracture	56 (38%)	36 (49%)	0.069
Sternal fracture	12 (8%)	7 (10%)	0.800
Clavicle fracture	34 (23%)	12 (16%)	0.294
Scapula fracture	33 (22%)	12 (16%)	0.376
Pneumothorax	114 (77%)	49 (67%)	0.143
Hemothorax	82 (55%)	34 (47%)	0.253
No. rib fractures	12 (7–15)	10 (7–15)	0.353
≥1 Bicortically displaced rib fractures	126 (85%)	56 (77%)	0.136
Bilateral rib fractures	72 (49%)	38 (52%)	0.669
Flail segment or flail chest	91 (61%)	37 (51%)	0.148
RibScore	3.5 (2–5)	3 (2–4)	0.144
Thoracostomy tube placed before admission CT	44 (30%)	18 (25%)	0.525
Hours between injury and admission CT	1:50 (1:22–3:06)	1:44 (1:15–3:53)	0.761
BPC18 on admission CT	4 (3–6)	4 (2–5)	0.144
Grouped pulmonary contusion severity No pulmonary contusion present	3 (2%)	2 (3%)	0.414
Mild contusion (BPC18 scores 1–3)	52 (35%)	28 (38%)	
Moderate contusion (BPC18 scores 4–6)	59 (40%)	33 (45%)	
Severe contusion (BPC18 scores 7–18)	34 (23%)	10 (14%)	

Data are shown as median (P_25_–P_75_) or as n (%). Significant differences are printed in bold.

BMI, body mass index; COPD, chronic obstructive pulmonary disease; CT, computed tomography; MCC, motor cycle crash; MVC, motor vehicle collision.

Pulmonary contusion severity as expressed by BPC18 was associated with a higher likelihood of pneumonia (odds ratio [OR], 1.15 [95% confidence interval (CI), 1.01–1.31]), need for tracheostomy (OR, 1.23 [95% CI, 1.07–1.41]), and need for mechanical ventilation (OR, 1.22 [95% CI, 1.22 [1.09–1.38]). Also, BPC18 was associated with longer intensive care unit (ICU) length of stay (unadjusted *β*, 1.00 [95% CI, 0.56–1.45]), more mechanical ventilation days (unadjusted *β*, 0.90 [95% CI, 0.30–1.50]), and longer hospital length of stay (unadjusted *β*, 1.45 [95% CI, 0.43–2.46]).

Differences were found in outcomes for SSRF compared with nonoperatively managed patients, stratified for pulmonary contusion severity (Table 2). Surgical stabilization of rib fracture patients with mild pulmonary contusions had better respiratory outcomes and needed fewer ICU days, compared with patients who underwent nonoperative management. Surgical stabilization of rib fracture patients with moderate pulmonary contusions had fewer mechanical ventilation days compared with patients who underwent nonoperative management. No differences in outcomes were found between SSRF patients and nonoperatively managed patients when they had severe pulmonary contusions.

**TABLE 2:** Comparison of Outcomes Between Patients Undergoing SSRF Versus Nonoperative Treatment, Stratified for Pulmonary Contusion Severity

	Mild Contusion n = 80		*p*	Moderate Contusion n = 92		*p*	Severe Contusion n = 44		*p*
	SSRF	Non-op		SSRF	Non-op		SSRF	Non-op	
Pneumonia	**4 (8%)**	**8 (29%)**	**0.020**	7 (12%)	8 (24%)	0.147	8 (24%)	3 (30%)	0.692
Tracheostomy*	7 (14%)	6 (25%)	0.327	10 (18%)	7 (28%)	0.381	12 (39%)	4 (50%)	0.694
Mortality	0 (0%)	1 (4%)	0.350	0 (0%)	1 (3%)	1.000	0 (0%)	1 (3%)	1.000
ICU admission	48 (92%)	26 (93%)	1.000	56 (95%)	32 (97%)	1.000	34 (100%)	10 (100%)	1.000
ICU LOS	**4 (2–6)**	**5 (3–15)**	**0.041**	5 (2–11)	7 (3–16)	0.130	6 (4–15)	13 (6–20)	0.143
MV need	13 (25%)	12 (43%)	0.131	27 (46%)	21 (64%)	0.129	20 (59%)	6 (60%)	1.000
MV days	**0 (0–2)**	**0 (0–13)**	**0.047**	**0 (0–6)**	**4 (0–19)**	**0.036**	3 (0–12)	9 (0–24)	0.481
Hospital LOS	9 (5–15)	12 (6–18)	0.147	11 (7–19)	14 (8–24)	0.128	13 (8–26)	18 (12–39)	0.273

*Patients with head AIS score of >3 are excluded from this analysis with total n = 198.

Data are shown as median (P_25_–P_75_) or as n (%). Significant differences are printed in bold.

Mild contusion is BPC18 scores of 1 to 3, moderate contusion is BPC18 scores of 4 to 6, and severe contusion is BPC18 scores of 7 to 18.

LOS, length of stay; MV, mechanical ventilation; non-op, nonoperative management of rib fractures.

To evaluate the association between SSRF and outcomes, we adjusted for injury severity using regression analyses (Table 3). These multivariable regressions indicated that, after adjusting for injury severity, SSRF patients with mild pulmonary contusions had a shorter stay in the ICU compared with nonoperatively managed patients with mild contusions (adjusted *β*, −2.51 [95% CI, −4.87 to −0.16]). Similarly, after adjustment, SSRF patients with moderate pulmonary contusions had fewer days on mechanical ventilation (adjusted *β*, −5.19 [95% CI, −10.2 to −0.17]) compared with nonoperatively managed patients with moderate pulmonary contusions. In the adjusted analyses for patients with severe pulmonary contusions, no differences in in-hospital outcomes were found between SSRF versus nonoperatively managed patients.

**TABLE 3:** Association Adjusted for Concomitant Injuries Between SSRF and Clinical Outcomes

	Mild Pulmonary Contusion		Moderate Pulmonary Contusion		Severe Pulmonary Contusion	
	n	Adjusted OR (95% CI)	n	Adjusted OR (95% CI)	n	Adjusted OR (95% CI)
Pneumonia non-op	28	Reference	33	Reference	10	Reference
SSRF	52	0.31 (0.08–1.28)	59	0.48 (0.15–1.57)	34	1.25 (0.21–7.28)
Tracheostomy* non-op	24	Reference	25	Reference	8	Reference
SSRF	51	0.56 (0.16–2.02)	55	0.45 (0.14–1.51)	31	0.90 (0.17–4.77)
Mortality non-op	28	Reference	33	Reference	10	Reference
SSRF	52	0.00 (0.00–**)	59	0.00 (0.00–**)	34	0.00 (0.00–**)
ICU admission non-op	28	Reference	33	Reference	10	Reference
SSRF	52	1.21 (0.20–7.47)	59	0.62 (0.06–6.35)	34	0.00 (0.00–**)
MV need non-op	28	Reference	33	Reference	10	Reference
SSRF	52	0.61 (0.21–1.74)	59	0.48 (0.18–1.33)	34	1.32 (0.28–6.12)
		**Adjusted *β* (95% CI)**		**Adjusted *β* (95% CI)**		**Adjusted *β* (95% CI)**
ICU LOS non-op	28	Reference	33	Reference	10	Reference
SSRF	52	**−2.51 (−4.87 to −0.16)**	59	−1.72 (−4.97 to 1.54)	34	−0.53 (−8.52 to 7.46)
MV days non-op	28	Reference	33	Reference	10	Reference
SSRF	52	−2.58 (−5.53 to 0.37)	59	**−5.19 (−10.2 to −0.17)**	34	−0.75 (−10.1 to 8.63)
Hospital LOS non-op	28	Reference	33	Reference	10	Reference
SSRF	52	−2.36 (−10.13 to 8.63)	59	−7.35 (−17.42 to 2.71)	34	−2.09 (−16.1 to 11.9)

*Patients with head AIS score of >3 are excluded from this analysis.

**Indicates an infinite number.

Adjusted for ISS. Significant associations are printed in bold.

Abd, abdomen; *β*, beta coefficient; CI, confidence interval; LOS, length of stay; MV, mechanical ventilation.

## DISCUSSION

This study aimed to evaluate the association between pulmonary contusion severity and outcomes after SSRF. We found that pulmonary contusion severity, as measured by BPC18, was associated with worse respiratory outcomes, and longer ICU and hospital length of stay. In patients with pulmonary contusions, SSRF was not associated with worse outcomes, even when adjusted for injury severity. Moreover, SSRF might be associated with better outcomes for patients with mild to moderate pulmonary contusions.

Some recent studies have suggested that SSRF for patients with pulmonary contusions is safe and effective, which aligns with our results.^23–25^ The finding that outcomes did not differ for SSRF versus nonoperatively managed patients with severe pulmonary contusions suggests that those severe contusions might negate the benefits of SSRF, as previously has been described.^18^ Specifically, pulmonary morbidity such as pneumonia, respiratory failure, and tracheostomy may be driven primarily by the severe contusion in this group rather than the chest wall injury. Potentially, SSRF could mitigate worsening of mild to moderate pulmonary contusions and thereby lead to better clinical outcomes, although this was not demonstrated with our data. Importantly, our results do not support previously stated recommendations that pulmonary contusions are a contraindication to SSRF.^19,20^

This is the first study specifically evaluating the clinical outcomes of SSRF in association with pulmonary contusion severity. In addition, a strength of this study is that it accounted for varying degrees of severity of pulmonary contusions in a standardized way on chest CT. Chest CT is highly sensitive to identify pulmonary contusions and is predictive for the need for mechanical ventilation, which, in contrast, is limited when using chest x-rays only.^10^

However, to quantify pulmonary contusion severity, multiple methods have been described, mostly based on the volume of the contused lung, but no universal classification currently exists.^15,26–28^ Although chest CT is highly sensitive for diagnosing pulmonary contusion even in presence of pneumothorax or pleural fluid, evaluating contusion severity can be challenging.^12^ Interobserver variability exists for scoring BPC18, which is a limitation of this study. In addition, pulmonary contusions evolve over time and can worsen in the hours after injury.^10,16^ Relying on admission CT only could have caused underestimation of the extent of pulmonary contusions potentially leading to respiratory failure.

Moreover, because of the retrospective nature of the study, comparing the SSRF and nonoperatively managed patients is subject to bias. Although the analysis was adjusted for injury severity by ISS, residual bias by other unmeasured patient or injury characteristics cannot be ruled out. Therefore, the association between SSRF and outcomes for patients with pulmonary contusions in addition to severe rib fractures should be interpreted cautiously because causation cannot be proven with this retrospective cohort. Last, only clinical outcomes were evaluated; for example, biomarkers of systemic inflammation might provide more objective evidence. Multiple rib fractures are related to impaired quality of life, both short-term and long-term.^4–6^ Consequently, patient-reported quality of life outcomes are at least as important as clinical outcomes for evaluating the effectiveness and safety of SSRF in presence of pulmonary contusions. Future SSRF studies accounting for pulmonary contusion severity are needed to evaluate these missing quality of life outcomes.

In conclusion, our results suggest that pulmonary contusions are not a contraindication to SSRF, regardless of the severity of the contusion. On the contrary, SSRF might be of benefit to clinical outcomes, especially in presence of mild to moderate pulmonary contusions.

## Supplementary Material

SUPPLEMENTARY MATERIAL
