# Supplementary material for: Rib fixation in patients with severe rib fractures and pulmonary contusions: Is it safe?
Source: J Trauma Acute Care Surg. 2022 Sep 19;93(6):721–6. doi: 10.1097/TA.0000000000003790 (PMC9671593; doi:10.1097/TA.0000000000003790)
Supplement: SUPPLEMENTARY MATERIAL [file jt-93-721-s001.docx]

# **Background**

Chest wall trauma is prevalent and morbid. Rib fractures account for around 10% of all trauma admissions and are associated with increased mortality and morbidity, and decreased quality of life.(1-6) Severe rib fracture patterns are often accompanied by other injuries, especially pulmonary contusions.(7, 8) Pulmonary contusions are documented in over 50% of patients with a flail chest.(9) Pulmonary contusions have dynamic pathophysiology which, depending on the extent of the injury, can lead to pneumonia, acute respiratory distress syndrome (ARDS), and mortality.(8, 10, 11)

Currently, chest computerized tomography (CT) is the recommended diagnostic modality, since it is highly sensitive in diagnosing pulmonary contusions.(12, 13) Because pulmonary contusions can vary in size and severity, dichotomously classifying the presence or absence of pulmonary contusions is insufficient to appreciate the extent of the injury.(14) Furthermore, the severity of the pulmonary contusions detected on CT-scan can change radiographically over time, especially in the first hours after injury. (15, 16)

Surgical stabilization of rib fractures (SSRF) is increasingly used in the management of patients with severe rib fractures.(17) Pulmonary contusions have traditionally been considered a relative contraindication to SSRF because the pulmonary morbidity is presumed to arise predominantly from the contusion as opposed to the rib fractures.(18-20) Moreover, pulmonary contusion may increase the risk of general anesthesia and SSRF. However, there is a paucity of published data specifically about SSRF concerning the presence and severity of pulmonary contusions.

This study aimed to evaluate the association between pulmonary contusion severity and outcomes after SSRF. We hypothesized that outcomes would be worse in patients who undergo SSRF compared to patients whose rib fractures are managed non-operatively.

# **Methods**

*Setting and study population*

We retrospectively analyzed trauma patients who were admitted with three or more displaced (≥ 50% cortical displacement on axial CT imaging) rib fractures or flail segment (two or more consecutive ribs with fractures in two or more locations) from our prospectively maintained database in a Level 1 Trauma Center. We included adult (≥18 years) patients from October 2010 to October 2021 if a chest CT was conducted on the first day of admission. Approval from the Institutional Review Board was obtained. The STROBE guideline was used to ensure proper reporting of methods, results, and discussion (SDC 1, <http://links.lww.com/TA/C702>).

*Variables*

Clinical data were retrieved from the patient’s medical records, including age, sex, past medical history, time and mechanism of injury, abbreviated injury score (AIS) and injury severity score (ISS), injury characteristics, and surgical procedures. Similarly, clinical outcomes were retrieved. The primary outcome variable studied was pneumonia rate. Secondary outcomes included rates of tracheostomy, mortality, mechanical ventilation days, and intensive care and hospital length of stay. All patients requiring mechanical ventilation were placed on a standard protocol including lung protective (6-8ml/kg ideal weight) ventilation. Patients with severe head injury as defined by head AIS greater than three were excluded from the analysis evaluating the tracheostomy rate. Patients were evaluated on the admission chest-CT for the presence of hemothorax, pneumothorax, bilateral rib fractures, flail segment, the total number of rib fractures, RibScore, and fracture displacement (undisplaced, offset, displaced) as defined by the CWIS Taxonomy.(21, 22)

*Pulmonary contusions*

The presence and severity of pulmonary contusions were evaluated on the admission chest CT in axial and coronal views in the lung window on a maximum slice thickness of 3.5 mm. Pulmonary contusion severity was quantified using the Blunt Pulmonary Contusion 18 score (BPC18). (15) In this score, the lung fields are divided into an upper, middle, and lower third, and for each third, a score of one to three was assigned. A score of one corresponds with mild contusion with up to 33% opacification of the field, a score of two is a moderate contusion with 33% to 66% opacification, and a score of three corresponds to severe contusion with over 66% opacification. The scores are summed, resulting in a maximum score of nine per lung, and a maximum total score of 18. All chest CTs were reviewed independently for BPC18 by at least two observers. One of the observers was a physician who reviewed and scored all chest CTs. The second independent BPC18 score was assigned either by another physician or a research coordinator who was trained by the other physicians to score BPC18. Cases with more than three points difference were reviewed again within the research team to reach consensus on the score. The BPC18 score assigned by the observer who scored all chest CTs was used when discrepancies of less than three points occurred. Pulmonary contusion severity was defined as mild with BPC18 of 1-3, moderate with 4-6, and severe with 7-18. An example of a mild, moderate, and severe pulmonary contusion is shown in Figure 1.

*Statistical analysis*

Data were analyzed using the Statistical Package for the Social Sciences (SPSS) version 28 (SPSS, Chicago, III., USA). The normality of continuous data was tested with the Shapiro-Wilk test. All continuous variables except age were nonparametric and are presented as median with percentiles. Categorical variables are presented as frequencies and percentages. Missing values were not replaced. Patients were divided into those who underwent SSRF versus those managed non-operatively and stratified for pulmonary contusion severity. Comparisons were made using the independent t-test and chi-square test for normally distributed data and the Mann-Whitney U and Fisher’s exact tests for nonparametric data. Adjustment for confounding by concomitant injury was done with logistic regression analyses for the association between SSRF and categorical outcomes and linear regression for the continuous clinical outcomes. P-values were considered significant if below 0.05.

# **Results**

A total of 221 patients were included and SSRF was performed in 148 (67%). The mean age was 52 years in both with SSRF and non-operatively managed patients (p=0.812). Other baseline patient characteristics were also comparable between groups (Table 1). Non-operatively managed patients had a similar chest injury severity to SSRF patients; both had a median chest abbreviated injury score (AIS) of 3 (P_25_-P_75_ 3-4; p=0.479). However, the non-operatively managed patients were overall more severely injured with a trend towards a higher injury severity score (ISS) (median 24 (P_25_-P_75_ 14-35) vs. 21 (P_25_-P_75_ 17-27), p=0.068), and a significantly higher head AIS (median 0 (P_25_-P_75_ 0-3) vs. 0 (P_25_-P_75_ 0-2) p=0.017) and abdomen/pelvis AIS (median 2 (P_25_-P_75_ 0-3) vs. 0 (P_25_-P_75_ 0-2), p=0.003) compared to patients who underwent SSRF. The median time between injury and admission CT was just below 2 hours in both groups (p=0.761). BPC18 on admission CT was 4 (P_25_-P_75_ 2-5 and P_25_-P_75_ 3-6; p=0.144) in both non-operatively managed and SSRF patients. Among the SSRF group, the median time to surgery was 1 day (P_25_-P_75_ 0-2).

Pulmonary contusion severity as expressed by BPC18 was associated with a higher likelihood of pneumonia (OR 1.15 (95%CI 1.01-1.31)), need for tracheostomy (OR 1.23 (95% CI 1.07-1.41), and need for mechanical ventilation (OR 1.22 (95%CI 1.22 (1.09-1.38)). Also BPC18 was associated with longer intensive care unit length of stay (unadjusted B 1.00 (95% CI 0.56;1.45)), more mechanical ventilation days (unadjusted B 0.90 (95% CI 0.30;1.50)), and longer hospital length of stay (unadjusted B 1.45 (95% CI 0.43;2.46)).

Differences were found in outcomes for SSRF compared to non-operatively managed patients, stratified for pulmonary contusion severity (Table 2). SSRF patients with mild pulmonary contusions had better respiratory outcomes and needed fewer ICU days, compared to patients who underwent non-operative management. SSRF patients with moderate pulmonary contusions had fewer mechanical ventilation days compared to patients who underwent non-operative management. No differences in outcomes were found between SSRF patients and non-operatively managed patients when they had severe pulmonary contusions.

To evaluate the association between SSRF and outcomes, we adjusted for injury severity using regression analyses (Table 3). These multivariable regressions indicated that after adjusting for injury severity, SSRF patients with mild pulmonary contusions had a shorter stay in the ICU compared to non-operatively managed patients with mild contusions (adjusted B -2.51 (95%CI -4.87;-0.16)). Similarly, after adjustment, SSRF patients with moderate pulmonary contusions had fewer days on mechanical ventilation (adjusted B -5.19 (95%CI -10.2;-0.17)) compared to non-operatively managed patients with moderate pulmonary contusions. In the adjusted analyses for patients with severe pulmonary contusions, no differences in in-hospital outcomes were found between SSRF versus non-operatively managed patients.

# **Discussion**

This study aimed to evaluate the association between pulmonary contusion severity and outcomes after SSRF. We found that pulmonary contusion severity, as measured by BPC18, was associated with worse respiratory outcomes, and longer ICU and hospital length of stay. In patients with pulmonary contusions, SSRF was not associated with worse outcomes, even when adjusted for injury severity. Moreover, SSRF might be associated with better outcomes for patients with mild to moderate pulmonary contusions.

Some recent studies have suggested that SSRF for patients with pulmonary contusions is safe and effective, which aligns with our results.(23-25) The finding that outcomes did not differ for SSRF versus non-operatively managed patients with severe pulmonary contusions, suggests that those severe contusions might negate the benefits of SSRF, as previously has been described.(18) Specifically, pulmonary morbidity such as pneumonia, respiratory failure, and tracheostomy may be driven primarily by the severe contusion in this group rather than the chest wall injury. Potentially, SSRF could mitigate worsening of mild to moderate pulmonary contusions and thereby lead to better clinical outcomes, although this was not demonstrated with our data. Importantly, our results do not support previously stated recommendations that pulmonary contusions are a contraindication to SSRF.(19, 20)

This is the first study specifically evaluating the clinical outcomes of SSRF in association with pulmonary contusion severity. Additionally, a strength of this study is that it accounted for varying degrees of severity of pulmonary contusions in a standardized way on chest CT. Chest CT is highly sensitive to identify pulmonary contusions and is predictive for the need for mechanical ventilation, which, in contrast, is limited when using chest x-rays only.(10)

However, to quantify pulmonary contusion severity, multiple methods have been described, mostly based on the volume of the contused lung, but no universal classification currently exists.(15, 26-28) Although chest CT is highly sensitive for diagnosing pulmonary contusion even in presence of pneumothorax or pleural fluid, evaluating contusion severity can be challenging.(12) Interobserver variability exists for scoring BPC18, which is a limitation of this study. In addition, pulmonary contusions evolve over time and can worsen in the hours after injury(10, 16). Relying on admission CT only, could have caused underestimation of the extent of pulmonary contusions potentially leading to respiratory failure.

Moreover, due to the retrospective nature of the study, comparing the SSRF and non-operatively managed patients is subject to bias. Although the analysis was adjusted for injury severity by ISS, residual bias by other unmeasured patient or injury characteristics cannot be ruled out. Therefore, the association between SSRF and outcomes for patients with pulmonary contusions in addition to severe rib fractures should be interpreted cautiously because causation cannot be proven with this retrospective cohort. Last, only clinical outcomes were evaluated; e.g. biomarkers of systemic inflammation might provide more objective evidence. Multiple rib fractures are related to impaired quality of life, both short-term and long-term.(4-6) Consequently, patient-reported quality of life outcomes are at least as important as clinical outcomes for evaluating the effectiveness and safety of SSRF in presence of pulmonary contusions. Future SSRF studies accounting for pulmonary contusion severity are needed to evaluate these missing quality of life outcomes.

In conclusion, our results suggest that pulmonary contusions are not a contraindication to SSRF, regardless of the severity of the contusion. On the contrary, SSRF might be of benefit to clinical outcomes, especially in presence of mild to moderate pulmonary contusions.

# **Supplemental digital content**

**SDC 1** STROBE Checklist of items that should be included in reports of observational studies

# **Table 1.** Baseline and injury characteristics of patients who underwent surgical stabilization of rib fractures, compared to patients who were managed non-operatively

|  | SSRF  N=148 | Non-operative  N=73 | p-value |
| --- | --- | --- | --- |
| Age, years, mean (SD) | 52 (16) | 52 (16) | 0.812 |
| Male | 103 (70%) | 52 (71%) | 0.876 |
| Comorbidities Any | 70 (47%) | 37 (51%) | 0.669 |
| Asthma | 12 (8%) | 4 (5%) | 0.588 |
| COPD | 6 (4%) | 4 (5%) | 0.733 |
| Diabetes | 19 (13%) | 8 (11%) | 0.828 |
| Chronic kidney disease | 0 (0%) | 1 (1%) | 0.330 |
| Chronic heart failure | 1 (1%) | 0 (0%) | 0.670 |
| Current smoker | 47 (32%) | 28 (38%) | 0.366 |
| BMI | 26 (23-30) | 27 (24-31) | 0.118 |
| Injury mechanism MVC/MCC | 60 (41%) | 33 (45%) | 0.145 |
| Auto vs. pedestrian | 27 (18%) | 12 (16%) |  |
| Auto vs. bike or ski- accident | 29 (20%) | 8 (11%) |  |
| Fall | 29 (20%) | 16 (22%) |  |
| Crush injury | 2 (1%) | 0 (0%) |  |
| Other or unknown | 1 (1%) | 4 (5%) |  |
| ISS | 21 (17-27) | 24 (14-35) | 0.068 |
| AIS **Head/neck** | **0 (0-2)** | **0 (0-3)** | **0.017** |
| Face | 0 (0-0) | 0 (0-0) | 0.769 |
| Chest | 3 (3-4) | 3 (3-4) | 0.479 |
| **Abdomen/pelvis** | **0 (0-2)** | **2 (0-3)** | **0.003** |
| Extremities | 2 (0-2) | 2 (0-2) | 0.342 |
| External | 1 (0-1) | 1 (0-1) | 0.578 |
| Isolated thoracic injury | 14 (9%) | 3 (4%) | 0.190 |
| Spinal fracture | 56 (38%) | 36 (49%) | 0.069 |
| Sternal fracture | 12 (8%) | 7 (10%) | 0.800 |
| Clavicle fracture | 34 (23%) | 12 (16%) | 0.294 |
| Scapula fracture | 33 (22%) | 12 (16%) | 0.376 |
| Pneumothorax | 114 (77%) | 49 (67%) | 0.143 |
| Hemothorax | 82 (55%) | 34 (47%) | 0.253 |
| Number of rib fractures | 12 (7-15) | 10 (7-15) | 0.353 |
| ≥1 Bicortically displaced rib fractures | 126 (85%) | 56 (77%) | 0.136 |
| Bilateral rib fractures | 72 (49%) | 38 (52%) | 0.669 |
| Flail segment or flail chest | 91 (61%) | 37 (51%) | 0.148 |
| RibScore | 3.5 (2-5) | 3 (2-4) | 0.144 |
| Thoracostomy tube placed before admission CT | 44 (30%) | 18 (25%) | 0.525 |
| Hours between injury and admission CT | 1:50 (1:22-3:06) | 1:44 (1:15-3:53) | 0.761 |
| BPC18 on admission CT | 4 (3-6) | 4 (2-5) | 0.144 |
| Grouped pulmonary contusion severity  No pulmonary contusion present | 3 (2%) | 2 (3%) | 0.414 |
| Mild contusion (BPC18 1-3) | 52 (35%) | 28 (38%) |  |
| Moderate contusion (BPC18 4-6) | 59 (40%) | 33 (45%) |  |
| Severe contusion (BPC18 7-18) | 34 (23%) | 10 (14%) |  |

Data are shown as median (P_25_-P_75_) or as N (%). Significant differences are printed in bold.

AIS, abbreviated injury score; BMI, body mass index; BPC18, blunt pulmonary contusion score-18; COPD, chronic obstructive pulmonary disease; CT, computed tomography; ISS, injury Severity Score; MCC, motor cycle crash; MVC, motor vehicle collision; SSRF, surgical stabilization of rib fractures

# **Table 2.** Comparison of outcomes between patients undergoing surgical stabilization of rib fractures versus non-operative treatment, stratified for pulmonary contusion severity

|  | Mild contusion  N=80 | | p-value | Moderate contusion  N=92 | | p-value | Severe contusion  N=44 | | p-value |
| --- | --- | --- | --- | --- | --- | --- | --- | --- | --- |
|  | SSRF | Non-op |  | SSRF | Non-op |  | SSRF | Non-op |  |
| Pneumonia | **4 (8%)** | **8 (29%)** | **0.020** | 7 (12%) | 8 (24%) | 0.147 | 8 (24%) | 3 (30%) | 0.692 |
| Tracheostomy* | 7 (14%) | 6 (25%) | 0.327 | 10 (18%) | 7 (28%) | 0.381 | 12 (39%) | 4 (50%) | 0.694 |
| Mortality | 0 (0%) | 1 (4%) | 0.350 | 0 (0%) | 1 (3%) | 1.000 | 0 (0%) | 1 (3%) | 1.000 |
| ICU-admission | 48 (92%) | 26 (93%) | 1.000 | 56 (95%) | 32 (97%) | 1.000 | 34 (100%) | 10 (100%) | 1.000 |
| ICU-LOS | **4 (2-6)** | **5 (3-15)** | **0.041** | 5 (2-11) | 7 (3-16) | 0.130 | 6 (4-15) | 13 (6-20) | 0.143 |
| MV-need | 13 (25%) | 12 (43%) | 0.131 | 27 (46%) | 21 (64%) | 0.129 | 20 (59%) | 6 (60%) | 1.000 |
| MV-days | **0 (0-2)** | **0 (0-13)** | **0.047** | **0 (0-6)** | **4 (0-19)** | **0.036** | 3 (0-12) | 9 (0-24) | 0.481 |
| Hospital-LOS | 9 (5-15) | 12 (6-18) | 0.147 | 11 (7-19) | 14 (8-24) | 0.128 | 13 (8-26) | 18 (12-39) | 0.273 |

Data are shown as median (P_25_-P_75_) or as N (%). Significant differences are printed in bold.

Mild contusion is BPC18 of 1-3, moderate contusion is BPC18 4-6, severe contusion is BPC18 7-18.

*Patients with head AIS>3 are excluded from this analysis with total n=198.

BPC18, blunt pulmonary contusion score-18; ICU, intensive care unit; LOS, length of stay; MV, mechanical ventilation; Non-op, non-operative management of rib fractures; SSRF, surgical stabilization of rib fractures

# **Table 3.** Association adjusted for concomitant injuries between surgical stabilization of rib fractures and clinical outcomes

|  | Mild pulmonary contusion | | Moderate pulmonary contusion | | Severe pulmonary contusion | |
| --- | --- | --- | --- | --- | --- | --- |
|  | N | Adjusted OR (95% CI) | N | Adjusted OR (95% CI) | N | Adjusted OR (95% CI) |
| Pneumonia Non-op | 28 | Reference | 33 | Reference | 10 | Reference |
| SSRF | 52 | 0.31 (0.08;1.28) | 59 | 0.48 (0.15;1.57) | 34 | 1.25 (0.21;7.28) |
| Tracheostomy* Non-op | 24 | Reference | 25 | Reference | 8 | Reference |
| SSRF | 51 | 0.56 (0.16;2.02) | 55 | 0.45 (0.14;1.51) | 31 | 0.90 (0.17;4.77) |
| Mortality Non-op | 28 | Reference | 33 | Reference | 10 | Reference |
| SSRF | 52 | 0.00 (0.00; **) | 59 | 0.00 (0.00; **) | 34 | 0.00 (0.00; **) |
| ICU-admission Non-op | 28 | Reference | 33 | Reference | 10 | Reference |
| SSRF | 52 | 1.21 (0.20;7.47) | 59 | 0.62 (0.06;6.35) | 34 | 0.00 (0.00; **) |
| MV-need Non-op | 28 | Reference | 33 | Reference | 10 | Reference |
| SSRF | 52 | 0.61 (0.21;1.74) | 59 | 0.48 (0.18;1.33) | 34 | 1.32 (0.28;6.12) |
|  |  | Adjusted B (95% CI) |  | Adjusted B (95% CI) |  | Adjusted B (95% CI) |
| ICU-LOS Non-op | 28 | Reference | 33 | Reference | 10 | Reference |
| SSRF | 52 | **-2.51 (-4.87;-0.16)** | 59 | -1.72 (-4.97;1.54) | 34 | -0.53 (-8.52;7.46) |
| MV-days Non-op | 28 | Reference | 33 | Reference | 10 | Reference |
| SSRF | 52 | -2.58 (-5.53;0.37) | 59 | **-5.19 (-10.2;-0.17)** | 34 | -0.75 (-10.1;8.63) |
| Hospital-LOS Non-op | 28 | Reference | 33 | Reference | 10 | Reference |
| SSRF | 52 | -2.36 (-10.13;8.63) | 59 | -7.35 (-17.42;2.71) | 34 | -2.09 (-16.1;11.9) |

Adjusted for Injury Severity Score (ISS). Significant associations are printed in bold.

*Patients with head AIS>3 are excluded from this analysis

** Indicates an infinite number

Abd, abdomen; AIS, abbreviated injury score; B, beta coefficient; BPC18, blunt pulmonary contusion score-18; CI, confidence interval; ICU, intensive care unit; ISS, injury severity score; LOS, length of stay; MV, mechanical ventilation; N, number of patients; OR, odds ratio; SSRF, surgical stabilization of rib fractures

**References**

1. Ziegler DW, Agarwal NN. The morbidity and mortality of rib fractures. *J Trauma*. 1994;37(6):975-9.

2. Peek J, Beks RB, Hietbrink F, De Jong MB, Heng M, Beeres FJP, et al. Epidemiology and outcome of rib fractures: a nationwide study in the Netherlands. *Eur J Trauma Emerg Surg*. 2022;48(1):265-71.

3. Flagel BT, Luchette FA, Reed RL, Esposito TJ, Davis KA, Santaniello JM, et al. Half-a-dozen ribs: the breakpoint for mortality. *Surgery*. 2005;138(4):717-23; discussion 23-5.

4. Marasco S, Lee G, Summerhayes R, Fitzgerald M, Bailey M. Quality of life after major trauma with multiple rib fractures. *Injury*. 2015;46(1):61-5.

5. Choi J, Khan S, Hakes NA, Carlos G, Seltzer R, Jaramillo JD, et al. Prospective study of short-term quality-of-life after traumatic rib fractures. *J Trauma Acute Care Surg*. 2021;90(1):73-8.

6. Choi J, Khan S, Sheira D, Hakes NA, Aboukhater L, Spain DA. Prospective study of long-term quality-of-life after rib fractures. *Surgery*. 2021.

7. Simon B, Ebert J, Bokhari F, Capella J, Emhoff T, Hayward T, 3rd, et al. Management of pulmonary contusion and flail chest: an Eastern Association for the Surgery of Trauma practice management guideline. *J Trauma Acute Care Surg*. 2012;73(5 Suppl 4):S351-61.

8. Beshay M, Mertzlufft F, Kottkamp HW, Reymond M, Schmid RA, Branscheid D, et al. Analysis of risk factors in thoracic trauma patients with a comparison of a modern trauma centre: a mono-centre study. *World J Emerg Surg*. 2020;15(1):45.

9. Dehghan N, de Mestral C, McKee MD, Schemitsch EH, Nathens A. Flail chest injuries: a review of outcomes and treatment practices from the National Trauma Data Bank. *J Trauma Acute Care Surg*. 2014;76(2):462-8.

10. Cohn SM, Dubose JJ. Pulmonary contusion: an update on recent advances in clinical management. *World J Surg*. 2010;34(8):1959-70.

11. Cohn SM. Pulmonary contusion: review of the clinical entity. *J Trauma*. 1997;42(5):973-9.

12. Gou J, Jiang Z, Wang P, Wang L, Chen W, Fang X. Diagnostic value of multi-slice spiral CT scan in lung compression ratio of patients with pulmonary contusion complicated by pneumothorax or hydropneumothorax. *Am J Transl Res*. 2021;13(4):3004-9.

13. Dillon DG, Rodriguez RM. Screening performance of the chest X-ray in adult blunt trauma evaluation: Is it effective and what does it miss? *Am J Emerg Med*. 2021;49:310-4.

14. Choi J, Tennakoon L, You JG, Kaghazchi A, Forrester JD, Spain DA. Pulmonary contusions in patients with rib fractures: The need to better classify a common injury. *Am J Surg*. 2021;221(1):211-5.

15. Tyburski JG, Collinge JD, Wilson RF, Eachempati SR. Pulmonary contusions: quantifying the lesions on chest X-ray films and the factors affecting prognosis. *J Trauma*. 1999;46(5):833-8.

16. Wen MN, Zhao G, Zhang JY, Zhao YH. Clinical study on the changes of lung-specific proteins: CC16 after lung contusion. *Exp Ther Med*. 2017;14(3):2733-6.

17. Kane ED, Jeremitsky E, Pieracci FM, Majercik S, Doben AR. Quantifying and exploring the recent national increase in surgical stabilization of rib fractures. *J Trauma Acute Care Surg*. 2017;83(6):1047-52.

18. Voggenreiter G, Neudeck F, Aufmkolk M, Obertacke U, Schmit-Neuerburg KP. Operative chest wall stabilization in flail chest--outcomes of patients with or without pulmonary contusion. *J Am Coll Surg*. 1998;187(2):130-8.

19. de Moya M, Nirula R, Biffl W. Rib fixation: Who, What, When? *Trauma Surg Acute Care Open*. 2017;2(1):e000059.

20. de Campos JRM, White TW. Chest wall stabilization in trauma patients: why, when, and how? *J Thorac Dis*. 2018;10(Suppl 8):S951-S62.

21. Chapman BC, Herbert B, Rodil M, Salotto J, Stovall RT, Biffl W, et al. RibScore: A novel radiographic score based on fracture pattern that predicts pneumonia, respiratory failure, and tracheostomy. *J Trauma Acute Care Surg*. 2016;80(1):95-101.

22. Edwards JG, Clarke P, Pieracci FM, Bemelman M, Black EA, Doben A, et al. Taxonomy of multiple rib fractures: Results of the chest wall injury society international consensus survey. *J Trauma Acute Care Surg*. 2020;88(2):e40-e5.

23. Althausen PL, Shannon S, Watts C, Thomas K, Bain MA, Coll D, et al. Early surgical stabilization of flail chest with locked plate fixation. *J Orthop Trauma*. 2011;25(11):641-7.

24. Jiang Y, Wang X, Teng L, Liu Y, Wang J, Zheng Z. Comparison of the Effectiveness of Surgical Versus Nonsurgical Treatment for Multiple Rib Fractures Accompanied with Pulmonary Contusion. *Ann Thorac Cardiovasc Surg*. 2019;25(4):185-91.

25. Zhang Y, Tang X, Xie H, Wang RL. Comparison of surgical fixation and nonsurgical management of flail chest and pulmonary contusion. *Am J Emerg Med*. 2015;33(7):937-40.

26. Mahmood I, El-Menyar A, Younis B, Ahmed K, Nabir S, Ahmed MN, et al. Clinical Significance and Prognostic Implications of Quantifying Pulmonary Contusion Volume in Patients with Blunt Chest Trauma. *Med Sci Monit*. 2017;23:3641-8.

27. Miller C, Stolarski A, Ata A, Pfaff A, Nadendla P, Owens K, et al. Impact of blunt pulmonary contusion in polytrauma patients with rib fractures. *Am J Surg*. 2019;218(1):51-5.

28. Strumwasser A, Chu E, Yeung L, Miraflor E, Sadjadi J, Victorino GP. A novel CT volume index score correlates with outcomes in polytrauma patients with pulmonary contusion. *J Surg Res*. 2011;170(2):280-5.

# **Figure legends**

# **Figure 1.** Examples of patients with mild, moderate, and severe pulmonary contusions on axial views of the admission chest CT

A) upper left: mild contusion, B) upper right: moderate contusion, C) lower left: severe contusion.
